# Triangulation of measles vaccination data in the United Kingdom of Great Britain and Northern Ireland

**DOI:** 10.2471/BLT.18.229138

**Published:** 2019-08-23

**Authors:** Michael Edelstein, Joanne White, Antoaneta Bukasa, Vanessa Saliba, Mary Ramsay

**Affiliations:** a National Infection Service, Public Health England, 61 Colindale Avenue, London NW9 5EQ, England.

## Abstract

**Objective:**

To illustrate how data triangulation involving routine data sources can optimize data usage and provide insights into vaccine programme effectiveness by considering measles vaccination and disease incidence data in England.

**Methods:**

We obtained data on measles, mumps and rubella (MMR) vaccine coverage in birth cohorts from 1985 to 2016 from child health records and adjusted for under-ascertainment and catch-up campaigns. We assumed that the population had no natural immunity and that vaccine effectiveness was 95% for one dose and 99.75% for two doses. Vaccinations done outside the routine schedule and in people who entered England after the age of immunization were identified from primary care records. Measles susceptibility was defined as the percentage of individuals who were not immune despite all vaccination activities. We triangulated measles susceptibility and incidence data.

**Findings:**

Median susceptibility was 4.6% (range: 1.2–9.2). Among cohorts eligible for two MMR vaccine doses, those born between 1998 and 2004 were most susceptible. Measles incidence was highest in these cohorts. Data from primary care and child health records were comparable for cohorts after 2000, suggesting that little supplementary vaccination took place. For cohorts before 2000, primary care data quality was insufficient for accurately estimating coverage.

**Conclusion:**

Triangulating routine data on measles vaccination coverage and disease surveillance provided new insights into population immunity and helped identify vulnerable groups, which was useful for prioritizing public health actions to close gaps in immunity. This approach could be applied in any country that routinely records vaccine coverage and disease incidence.

## Résumé

**Objectif:**

Illustrer comment la triangulation des données incluant des sources de données ordinaires peut optimiser l'utilisation des données et donner une idée de l'efficacité du programme de vaccination en considérant des données relatives à la vaccination contre la rougeole et à l'incidence de la maladie en Angleterre.

**Méthodes:**

Nous avons obtenu des données sur la couverture vaccinale contre la rougeole, les oreillons et la rubéole dans les cohortes de naissances de 1985 à 2016 à partir des dossiers médicaux des enfants et nous les avons ajustées en fonction de la sous-détermination et des campagnes de rattrapage. Nous sommes partis du principe que la population n'avait pas d'immunité naturelle et que l'efficacité du vaccin était de 95% pour une dose et de 99,75% pour deux doses. Les vaccinations pratiquées en dehors du calendrier habituel et chez des personnes entrées en Angleterre après l'âge de vaccination ont été identifiées à partir des dossiers relatifs aux soins primaires. La sensibilité à la rougeole a été définie comme le pourcentage d'individus qui n'étaient pas immunisés malgré toutes les activités de vaccination. Nous avons triangulé les données relatives à la sensibilité à la rougeole et à l'incidence de la rougeole.

**Résultats:**

La sensibilité médiane était de 4,6% (étendue: 1,2-9,2). Parmi les cohortes pouvant bénéficier de deux doses de vaccins ROR, celles nées entre 1998 et 2004 étaient les plus sensibles. L'incidence de la rougeole était plus élevée dans ces cohortes. Les données tirées des dossiers relatifs aux soins primaires et des dossiers médicaux des enfants étaient comparables pour les cohortes ultérieures à 2000, ce qui laisse entendre que peu de vaccinations supplémentaires ont été pratiquées. Dans le cas des cohortes antérieures à 2000, la qualité des données sur les soins primaires était insuffisante pour permettre une estimation précise de la couverture.

**Conclusion:**

La triangulation des données ordinaires sur la couverture vaccinale antirougeoleuse et la surveillance de la maladie a jeté une lumière nouvelle sur l'immunité de la population et aidé à identifier les groupes vulnérables, ce qui a permis d'établir l'ordre de priorité des actions de santé publique destinées à combler les lacunes en matière d'immunité. Cette approche pourrait être appliquée dans tous les pays qui enregistrent systématiquement la couverture vaccinale et l'incidence de la maladie.

## Resumen

**Objetivo:**

Ilustrar cómo la triangulación de datos con fuentes rutinarias de datos puede optimizar el uso de los mismos y proporcionar información sobre la eficacia de los programas de vacunación al considerar la vacunación contra el sarampión y los datos de incidencia de la enfermedad en Inglaterra.

**Métodos:**

Se obtuvieron datos sobre la cobertura de la vacuna contra el sarampión, las paperas y la rubéola (SPR) en las cohortes de nacimiento de 1985 a 2016 a partir de los registros de salud infantil y se ajustaron para tener en cuenta las campañas de recuperación y de los errores por la falta de observaciones. Se asumió que la población no tenía inmunidad natural y que la efectividad de la vacuna era del 95 % para una dosis y del 99,75 % para dos dosis. Las vacunas realizadas fuera del horario de rutina y en personas que ingresaron a Inglaterra después de la edad de vacunación fueron identificadas en los registros de atención primaria. La susceptibilidad al sarampión se definió como el porcentaje de individuos que no eran inmunes a pesar de todas las actividades de vacunación. Se triangularon los datos de susceptibilidad e incidencia del sarampión.

**Resultados:**

La mediana de susceptibilidad fue de 4,6 % (rango: 1,2-9,2). Entre las cohortes elegibles para dos dosis de la vacuna triple viral, las que nacieron entre 1998 y 2004 fueron las más susceptibles. La incidencia de sarampión fue mayor en estas cohortes. Los datos de los registros de atención primaria y de salud infantil fueron comparables para las cohortes después de 2000, lo que sugiere que se realizó muy poca vacunación suplementaria. Para las cohortes anteriores a 2000, la calidad de los datos de atención primaria fue insuficiente para estimar con precisión la cobertura.

**Conclusión:**

La triangulación de los datos de rutina sobre la cobertura de la vacunación contra el sarampión y la vigilancia de las enfermedades proporcionó nueva información sobre la inmunidad de la población y ayudó a identificar a los grupos vulnerables, lo que resultó útil para establecer prioridades en las medidas de salud pública a fin de subsanar las deficiencias en materia de inmunidad. Este enfoque podría aplicarse en cualquier país que registre de forma rutinaria la cobertura de la vacuna y la incidencia de la enfermedad.

## ملخص

**الغرض:**

توضيح كيف يمكن لتخطيط البيانات الذي يتضمن مصادر بيانات روتينية، أن يؤدي إلى تحسين استخدام البيانات وتوفير رؤى ثاقبة حول فعالية برنامج اللقاح عن طريق وضع بيانات لقاح الحصبة وتكرار الإصابة بالمرض بإنجلترا في الاعتبار.

**الطريقة:**

لقد حصلنا على بيانات حول تغطية لقاح الحصبة والنكاف والحصبة الألمانية (MMR) في مجموعات المواليد من 1985 إلى 2016، من سجلات صحة الأطفال، وقمنا بتعديلها وفقاً لعدم التحقق وحملات المتابعة. وافترضنا أن السكان لم يكن لديهم مناعة طبيعية، وأن فعالية هذا اللقاح كانت 95% لجرعة واحدة، و99.75% لجرعتين. تم التعرف من خلال سجلات الرعاية الأولية على التطعيمات التي تمت خارج المخطط الروتيني، وعلى الأشخاص الذين دخلوا إلى إنجلترا بعد سن التمنيع. تم تعريف قابلية الإصابة بالحصبة على أنها النسبة المئوية للأفراد الذين لم يكونوا محصنين على الرغم من كل أنشطة التطعيم. قمنا بتخطيط بيانات قابلية الإصابة بالحصبة، والإصابة الفعلية بها.

**النتائج:**

كان متوسط قابلية الإصابة هو 4.6% (المدى: 1.2 إلى 9.2). من بين المجموعات المؤهلة للحصول على جرعات لقاح MMR، كانت المواليد بين عامي 1998 و2004 هي الأكثر عرضة للإصابة. كان معدل الإصابة بالحصبة هو الأعلى بين هذه المجموعات. كانت البيانات المستقاة من سجلات الرعاية الأولية وسجلات صحة الأطفال قابلة للمقارنة بالنسبة لمجموعات ما بعد عام 2000، مما يوضح أنه تم الحصول على تطعيم تكميلي بسيط. بالنسبة للمجموعات ما قبل عام 2000، كانت جودة بيانات الرعاية الأولية غير كافية لتقدير التغطية بشكل دقيق.

**الاستنتاج:**

أدى تخطيط البيانات الروتينية حول تغطية التطعيم ضد الحصبة ومراقبة الأمراض، إلى تقديم رؤى ثاقبة جديدة حول مناعة السكان، والمساعدة في تحديد المجموعات المعرضة للخطر، والتي كانت مفيدة لمنح الأولوية لإجراءات الصحة العامة لعلاج ثغرات المناعة. يمكن تطبيق هذا الأسلوب في أية دولة تقوم بشكل روتيني بتسجيل بيانات تغطية اللقاح ومعدل الإصابة بالمرض. ****

## 摘要

**目的:**

通过考虑英国的麻疹疫苗接种和疾病发病率数据来说明涉及常规数据源的数据三角互证如何优化数据使用情况并提供有关疫苗计划效果的见解。

**方法:**

我们从 1985 年至 2016 年的儿童健康记录中获得了出生队列中麻疹、腮腺炎和风疹 (MMR) 疫苗覆盖率的相关数据并根据归因不足和弥补活动进行了调整。我们假设该人群没有天然免疫力，并且一剂疫苗有效率为 95％，两剂疫苗有效率为 99.75％。在初级保健记录中确定了在常规计划之外以及在免疫接种年龄后进入英国的人群中进行的疫苗接种。麻疹易感率是指不论各种免疫活动，非免疫人口所占的百分比。我们对麻疹易感率和发病率数据进行了三角互证。

**结果:**

中位数易感率为 4.6％（范围：1.2-9.2）。在符合两种 MMR 疫苗剂量的队列中，1998 年至 2004 年间出生的人群最易感染。麻疹发病率在这些队列中最高。2000 年以后，来自初级保健和儿童健康记录的数据在队列中具有可比性，表明很少进行补充疫苗接种。对于 2000 年以前的队列，初级保健数据质量不足以准确估算覆盖率。

**结论:**

麻疹疫苗接种覆盖率和疾病监测的三角互证常规数据针对人群免疫力提供了新的见解并有助于识别易感群体，从而有利于优先考虑公共保健措施，以缩小免疫差距。这种方法可以应用于任何经常记录疫苗覆盖率和发病率的国家。****

## Резюме

**Цель:**

Проиллюстрировать, как триангуляция данных с применением стандартных источников данных может оптимизировать использование данных и помогает определять наиболее эффективные способы реализации программы прививок в контексте данных о вакцинации от кори и распространенности заболевания в Англии.

**Методы:**

Авторы рассмотрели данные об охвате вакциной от кори, краснухи и свинки (MMR) младенцев, родившихся в период с 1985 по 2016 год, которые получены из медицинских карточек детей, и скорректировали их с учетом недостаточного выявления и кампаний подчищающей иммунизации. Авторы исходили из предположения, что естественный иммунитет в популяции отсутствует и что эффективность вакцинации составляла 95% после однократной дозы и 99,75% после двух доз. Случаи вакцинации с нарушением обычного графика и вакцинации лиц, попавших в Англию позже установленного возраста иммунизации, идентифицировались по первичным медицинским документам. Подверженность заболеванию корью определялась как процентная доля лиц, которые не приобрели иммунитет, несмотря на вакцинацию. Авторы провели триангуляцию данных по частоте заболевания корью и подверженности этому заболеванию.

**Результаты:**

Медианная подверженность заболеванию составила 4,6% (диапазон: 1,2–9,2). Среди когорт, которым полагалось получить две дозы вакцины MMR, наиболее подвержены заболеванию были дети, рожденные в период с 1998 по 2004 год. В этих когортах распространенность кори была наивысшей. Данные первичных учреждений здравоохранения и детских медицинских карт для когорт лиц, родившихся после 2000 года, были сопоставимыми, что позволяет предположить, что дополнительная вакцинация почти не проводилась. Для когорт лиц, родившихся до 2000 года, качество данных первичных медицинских документов было недостаточным для точного определения охвата вакцинацией.

**Вывод:**

Триангуляция стандартных данных об охвате вакцинацией от кори и надзоре за распространенностью заболевания позволила сделать новые выводы об иммунном статусе популяции и помогла выявить подверженные риску группы, что было важно для расстановки приоритетов в общественном здравоохранении с целью принятия мер по ликвидации пробелов в иммунизации. Такой подход применим в любой стране, которая регулярно фиксирует охват населения вакцинацией и частоту возникновения соответствующих заболеваний.****

## Introduction

A considerable amount of immunization coverage and surveillance data are available nationally, regionally and globally.^1^ Often, however, these data could be better used to aid decision-making on national and subnational immunization programmes. Particularly where a disease is close to being eliminated and the remaining few percent of susceptible individuals are being targeted, the need for accurate data increases as vaccine coverage increases.^2^ Synthesizing data from two or more sources (i.e. data triangulation) is a pragmatic approach to optimizing the use of existing data, thereby improving data quality and gaining insights into the performance of vaccine programmes.^1^ In this study, we used the example of measles in England to illustrate how the triangulation of routine data sources, namely different sources on coverage of the combined measles, mumps and rubella (MMR) vaccine and measles incidence data, can help evaluate data quality and provide estimates of population immunity, which can be used to inform a national measles elimination strategy. As these data sources are available in most settings and for many diseases, with varying degrees of granularity and quality, our approach should be broadly replicable.

Measles is a viral infection transmitted by the respiratory route and one of the most contagious human diseases.^3^ In 2016, approximately 90 000 deaths were attributable to the disease globally,^4^ down from more than 550 000 in 2000 thanks to the accelerated roll-out of measles immunization programmes.^4^ The commitment to eliminate measles (and rubella) is an important part of global efforts to improve health and reduce inequality. To achieve and maintain elimination, the World Health Organization (WHO) recommends that countries attain 95% coverage with two doses of measles-containing vaccine by the time children are 5 years of age.^5^ Modelling suggests that measles can be eliminated in most contexts if the proportion of children younger than 5 years susceptible to measles is less than 15% and the proportion of susceptible individuals aged 5 years and older is less than 5%.^6^

In England, before measles vaccine was introduced in 1968, 160 000 to 800 000 cases of measles were notified and around 100 deaths from acute disease were recorded each year.^7^ After the combined MMR vaccine was introduced in 1988, coverage rapidly reached 90% and disease incidence fell to a very low level. In 1994, a large catch-up programme was undertaken with measles–rubella vaccine and in October 1996, a second MMR vaccine dose was added.^7^ However, in 1998 a British doctor published a now-discredited study suggesting a link between MMR vaccine and autism.^8^ The resulting intense media interest had a substantial impact on MMR vaccine coverage, which dropped to about 80% in the late 1990s and early 2000s and took many years to recover.^9^ Since this fall, several catch-up campaigns have been implemented to address gaps in population immunity. Measles cases continued to rise and in 2006, endemic transmission became re-established in the United Kingdom of Great Britain and Northern Ireland. The incidence peaked at 3.2 per 100 000 population in 2012 and decreased to 1.0 per 100 000 in 2016.^10^ The United Kingdom is committed to measles elimination and has developed a national strategy in line with the European Vaccine Action Plan 2015–2020.^11^^,^^12^ Since 2012, coverage for the first MMR vaccine dose in children aged 24 months has been consistently over 90%.^9^^,^^10^ In 2016 and 2017, coverage for the first dose in children aged 5 years reached 95% for the first time.^9^ Since 2016, imported cases of measles have led to several outbreaks, with some limited spread in the population, particularly among individuals who missed the MMR vaccine when they were younger and in undervaccinated communities.^13^ Overall, the incidence of measles in England quadrupled between 2017 and 2018.^14^

### Routine coverage and surveillance data in England

Vaccine coverage in England is estimated by two methods. The first uses data from local Child Health Information Systems, which provide data to the cover of vaccination evaluated rapidly (COVER) programme.^15^ The second involves an online platform called ImmForm that automatically extracts immunization data from approximately 95% of primary care facilities (i.e. general practices).^16^ The COVER programme is designed to collect data on coverage by specific target ages (i.e. by children’s second and fifth birthdays) and because it includes the entire population eligible for vaccination, provides the most accurate estimates of coverage at the time of data collection. As coverage is not routinely assessed again, COVER data will not accurately reflect the current status of a given birth cohort many years later. Vaccines given at an older age may not be recorded and neither the numerator nor denominator in the coverage calculation will include individuals who arrived in England after their fifth birthday. Nevertheless, COVER data are used for reporting to WHO on the WHO and United Nations Children's Fund’s (UNICEF’s) Joint Reporting Form. 

By contrast, ImmForm data represent vaccine coverage recorded by general practices at the time of data extraction and include anyone in a specific birth cohort who was registered with the practice at that time (i.e. 2017 to 2018 for our study). Consequently, the data should cover vaccinations given either through routine vaccination programmes, during national catch-up campaigns, opportunistically, or outside England to any individual of any age. The accuracy of ImmForm data depends on the quality and completeness of clinical coding at each facility, these characteristics are known to have the greatest influence on immunization data quality globally.^1^ In the United Kingdom, clinicians are legally required to report suspected measles cases to the public health services. In addition, national surveillance systems require all suspected cases to be confirmed by laboratories using either an immunoglobulin-M antibody test on a serum or oral fluid specimen or a polymerase chain reaction technique.^11^

### Data triangulation

Data triangulation involves the synthesis of two or more data sources with the aim of assisting programme planning and decision-making. The process can identify and address limitations in any single data source or data collection method. In addition, deeper insights can be achieved by examining complementary data and putting them into a broader context. A recent report from a WHO-commissioned expert group on immunization data quality recommended that data triangulation should become the default approach to data analysis and use in its Expanded Programme on Immunization.^1^

The aim of our study was to use the example of measles elimination in England to demonstrate how data sources that are routinely available as part of the Expanded Programme on Immunization can be synthesized to improve vaccination data quality and to generate new information, for example on measles susceptibility and gaps in immunity, that can help guide decision-making on vaccine policy.

## Methods

In calculating the proportion of the English population susceptible to measles, we assumed a vaccine effectiveness of 95% for one MMR vaccine dose and 99.75% for two doses.^17^^–^^19^ In addition, we assumed there was no natural immunity because the level of circulating disease in the country over the past 30 years was low.

To estimate coverage of routine immunization for each birth cohort between the year from April 1985 to March 1986 (i.e. 1985–1986) and the year from April 2015 to March 2016 (i.e. 2015–2016), we used COVER data on the first and second MMR vaccine doses. Data collected at children’s fifth birthdays were generally available for birth cohorts from 1992–1993 until 2012–2013. We used data collected at the second birthday for individuals in birth cohorts after 2012–2013, who were too young during our study period to have had coverage of two doses assessed at 5 years of age, and for individuals in birth cohorts before 1992–1993, who were born before the second dose was included in the vaccination schedule. We applied a 50% coverage underestimate correction factor to COVER estimates. This percentage was based on a study that examined the extent of underestimation by checking the vaccination status of individuals not recorded as vaccinated in Child Health Information Systems.^20^ Further, to consider the possibility that the 50% correction factor was too high, we examined the effect of a 25% correction factor in a sensitivity analysis.

**Fig. 1:**
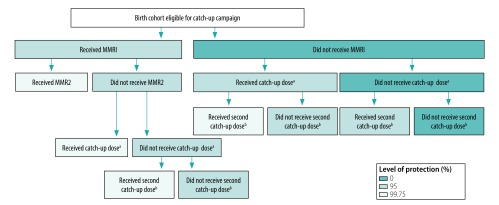
Flowchart for determining level of protection from measles in birth cohorts, England, 1985–2016

MMR1: first dose of measles, mumps and rubella vaccine; MMR2: second dose of measles, mumps and rubella vaccine.

^a^ For birth cohorts eligible for one catch-up campaign.

^b^ For birth cohorts eligible for more than one catch-up campaign.

Several birth cohorts included in the study were eligible for supplementary immunization in national catch-up campaigns (Table 1). Coverage data have been published for the 1994 and 2013 campaigns;^21^^,^^22^ for other campaigns, we used the best estimates from Public Health England (unpublished data). Fig. 1 describes how we determined the level of protection from measles among cohorts that were eligible for participation in catch-up campaigns. To ascertain the level of opportunistic vaccination after the routine vaccination age and outside of catch-up campaigns, we triangulated vaccine coverage data from the COVER programme (i.e. routine vaccination data only) with data from ImmForm, which potentially captures any vaccinations given up to the time of data extraction.

**Table 1:** Measles vaccination coverage and individuals susceptible to measles, by birth cohort, England, 1985–2016

Birth cohort^a^	Applicable catch-up campaign			Routine vaccination coverage,^c^ %			Adjusted routine vaccination coverage,^d^ %		Proportion susceptible to measles,^e^ %	Estimated no. of individuals in cohort in 2017	Estimated no. of susceptible individuals	Immunity level sufficient to interrupt transmission?^f^
	Name and date^b^	Coverage, %		First MMR vaccine dose	Second MMR vaccine dose		First MMR vaccine dose	Second MMR vaccine dose				
2015–2016	None	NA		91.2	NA		95.6	NA	9.2	674 807	61 947	Yes
2014–2015	None	NA		91.6	NA		95.8	NA	9.0	675 045	60 687	Yes
2013–2014	None	NA		91.4	NA		95.7	NA	9.1	682 356	61 992	Yes
2012–2013	None	NA		94.9	87.2		95.4	93.6	3.0	699 250	20 813	Yes
2011–2012	None	NA		95.0	87.6		97.5	93.8	2.9	721 708	21 070	Yes
2010–2011	None	NA		94.9	87.6		97.4	93.8	3.0	707 075	21 212	Yes
2009–2010	None	NA		94.6	88.4		97.3	94.2	3.1	694 480	21 529	Yes
2008–2009	None	NA		94.4	88.4		97.2	94.2	3.2	684 370	21 900	Yes
2007–2008	MMR 2008	Unknown (low)		94.2	88.3		97.1	94.1	3.3	689 769	22 762	Yes
2006–2007	MMR 2008	Unknown (low)		93.5	87.0		96.8	93.5	3.6	667 818	24 041	Yes
2005–2006	MMR 2008	Unknown (low)		92.4	84.6		96.2	92.3	4.2	654 366	27 483	Yes
2004–2005	MMR 2008	Unknown (low)		91.5	83.0		95.7	91.5	4.7	627 407	29 488	Yes
2003–2004	MMR 2008	Unknown (low)		89.9	80.1		94.9	90.1	5.5	616 975	33 934	No
2002–2003	MMR 2008 and 2013	Unknown (low) and 10.8		87.3	74.7		93.6	87.3	6.2	599 472	37 167	No
2001–2002	MMR 2008 and 2013	Unknown (low) and 10.8		86.8	73.2		93.4	86.6	6.4	589 606	37 735	No
2000–2001	MMR 2008 and 2013	Unknown (low) and 10.8		86.0	73.0		93.0	86.5	6.8	605 724	41 189	No
1999–2000	MMR 2008 and 2013	Unknown (low) and 10.8		88.6	74.0		94.3	87.0	5.7	623 262	35 526	No
1998–1999	MMR 2008 and 2013	Unknown (low) and 10.8		89.6	74.6		94.8	87.3	5.2	645 133	33 547	No
1997–1998	MMR 2008 and 2013	Unknown (low) and 10.8		90.5	74.6		95.3	87.3	4.8	657 005	31 536	Yes
1996–1997	MMR 2008 and 2013	Unknown (low) and 10.8		90.8	74.0		95.4	87.0	4.7	683 225	32 112	Yes
1995–1996	MMR 2008	Unknown (low)		91.7	74.2		95.9	87.1	4.8	689 511	33 097	Yes
1994–1995	MMR 2008	Unknown (low)		92.6	74.7		96.3	87.3	4.3	696 484	29 949	Yes
1993–1994	MMR 2008	Unknown (low)		93.5	76.4		96.8	88.2	3.9	725 250	28 285	Yes
1992–1993	MMR 2008	Unknown (low)		94.1	74.4		97.0	87.2	3.7	731 671	27 072	Yes
1991–1992	MMR2 1996 and MMR 2008	60 and unknown (low)		92.4	NA		96.2	NA	5.9	757 328	44 682	No
1990–1991	MMR2 1996 and MMR 2008	60 and unknown (low)		92.7	NA		96.4	NA	5.7	777 368	44 310	No
1989–1990	MMR2 1996 and MMR 2008	60 and unknown (low)		92.0	NA		96.0	NA	6.1	766 804	46 775	No
1988–1989	Measles–rubella 1994	92		89.8	NA		94.9	NA	1.2	760 183	9 122	Yes
1987–1988	Measles–rubella 1994	92		87.2	NA		93.6	NA	1.4	769 941	10 779	Yes
1986–1987	Measles–rubella 1994	92		90.8	NA		95.4	NA	1.2	750 908	9 011	Yes
1985–1986	Measles–rubella 1994	92		77.9	NA		88.9	NA	2.0	758 385	15 168	Yes
**Total**	NA	NA		ND	ND		ND	ND	4.6	21 382 686	975 920	NA

MMR: measles, mumps and rubella; MMR2: second dose of measles, mumps and rubella vaccine; NA: not applicable; ND: not determined.

^a^ Birth cohorts included individuals born between April in one year and March in the following year.

**Fig. 2:**
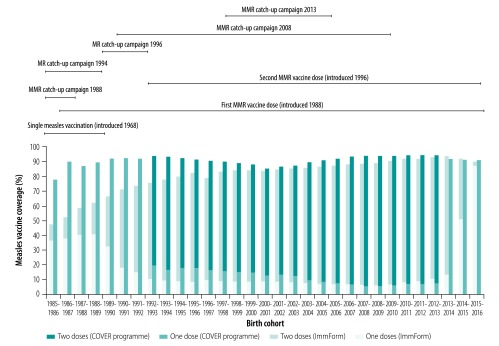
Measles vaccine coverage, by birth cohort, England, 1985–2016

COVER: cover of vaccination evaluated rapidly (programme); MMR: measles, mumps and rubella; MMR2: second dose of measles, mumps and rubella vaccine; MR: measles and rubella.

Notes: Estimates from the cover of vaccination evaluated rapidly (COVER) programme up to 1989 include coverage of both the single measles vaccine and the first measles, mumps and rubella (MMR) vaccine. For the COVER programme, coverage data were collected at the children’s second birthday for those in birth cohorts after 2012–2013 and before 1992–1993; otherwise data were collected at their fifth birthday. Birth cohorts included individuals born between April in one year and March in the following year.

^b^ Catch-up campaigns were carried out in 1998, 2008 and 2013 with measles, mumps and rubella (MMR) vaccine, in 1994 with measles–rubella vaccine and in 1996 with the second MMR vaccine dose (MMR2; Fig. 2).

^c^ Vaccine coverage was determined using data from local Child Health Information Systems, which provide data to the cover of vaccination evaluated rapidly (COVER) programme.

^d^ Adjusted vaccine coverage was calculated using a 50% coverage underestimate correction factor: for example, if the estimated proportion of the cohort not covered by vaccination was 8%, 4% (i.e. 50% of 8%) was added to the unadjusted coverage.

^e^ The susceptibility calculation took into account both routine and catch-up vaccination, as described in the methods.

^f^ The immunity level was regarded as sufficient to interrupt measles transmission when the reproductive number (*R_0_*) was under 1, which corresponded to an immunity level over 85% in children aged under 4 years and over 95% in those aged 5 years and over.

For each birth cohort, we calculated susceptibility (*S*) to measles, which was defined as the percentage of individuals in the birth cohort who had not been vaccinated or who were probably not immune despite routine, supplementary or opportunistic vaccination, using the equation: 
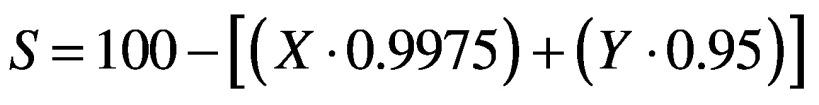
(1)Where *X* is the percentage of birth cohort who received ≥ 2 vaccine doses and *Y* is the percentage of birth cohort who received 1 dose. Then, using the size of the population in each age band in 2017 obtained from the Office of National Statistics,^23^ we calculated the number of susceptible individuals in the population and overall population susceptibility. Susceptibility in each birth cohort was compared to the target immunity level required to keep the reproductive number (*R_0_*) below one and, therefore, interrupt transmission in the population (*R_0_* is the number of additional cases each disease case generates in a susceptible population; if *R_0_* < 1, transmission will not carry on).^6^ Table 1 shows whether each cohort achieved this target or not. To further validate our susceptibility estimates, we compared the age-specific incidence of laboratory-confirmed cases of measles by year of diagnosis (restricted to cases with an onset between 2010 and 2018) with age-specific susceptibility. We focused on age-specific incidence and susceptibility rather than on annual incidence to identify reported cases that occurred in birth cohorts eligible for vaccination and to help us discover underprotected groups that could be targeted by practical changes to vaccination programmes (for example, by identifying appropriate age groups for a catch-up campaign).

All susceptibility calculations were performed using Microsoft Excel (Microsoft Corporation, Redmond, United States of America). All coverage and surveillance data were collected through national routine surveillance systems and the study was conducted using aggregated data as part of routine surveillance activities. No specific funding or formal ethical approval was required.

## Results

Table 1 shows coverage of the first and second MMR vaccine doses in birth cohorts between 1985–1986 and 2015–2016. The small differences between COVER and ImmForm estimates for birth cohorts between 2000–2001 and 2012–2013 (Fig. 2; ImmForm data at the children’s fifth birthday were not available after this date) suggest that little opportunistic vaccination took place after routine immunization and that no large groups of unvaccinated foreign-born children were registered with general practices in England. Since the quality of primary care data (i.e. ImmForm data) was low for birth cohorts before 2000–2001, coverage estimates were uncertain (Fig. 2).

**Fig. 3:**
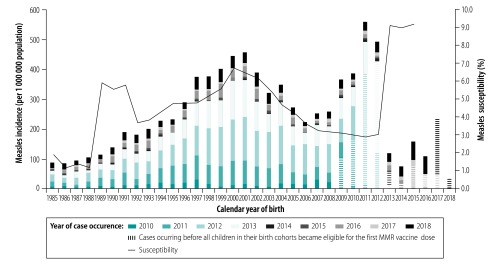
Measles susceptibility and incidence, by birth cohort, England, 1985–2018

Overall, measles susceptibility among people born between 1985 and 2016 was 4.6% (range: 1.2–9.2), which corresponds to 975 920 individuals in these birth cohorts in 2019 (Table 1). Of individuals who were eligible for the second MMR vaccine dose from October 1996 onwards, those born between 1998 and 2004 were in birth cohorts classified as not having a sufficiently high level of immunity to prevent measles transmission (Table 1). Surveillance data on measles cases confirmed that, among birth cohorts eligible for full vaccination, the incidence was highest in these cohorts (Fig. 3). Likewise, birth cohorts between 1989–1990 and 1991–1992 did not appear to achieve a sufficiently high level of immunity. However, high susceptibility in those cohorts was not matched by high disease incidence (Fig. 3), which indicates that coverage of the 1996 catch-up campaign was probably underestimated. The incidence of disease in individuals born in 2010 and 2011 was higher than that in more susceptible individuals born between 2000 and 2003 (Fig. 3). However, most cases in those born in 2010 and 2011 occurred before all children in their birth cohort became eligible for the first MMR vaccine dose (Fig. 3).

MMR: measles, mumps and rubella.

When a coverage underestimate correction factor of 25% rather than 50% was applied in the sensitivity analysis, susceptibility estimates suggested that no birth cohort between 1989–1990 and 2006–2007 achieved a sufficiently high level of immunity to prevent measles transmission. However, this apparently high susceptibility was not matched by a high observed disease incidence. Consequently, the correction factor of 50% was more likely to be correct.

## Discussion

Our application of data triangulation to measles vaccination in England, which considered data on vaccine coverage (including supplementary immunization) in individual birth cohorts and data on the age-specific incidence of measles, illustrates that the approach provides a pragmatic, simple and useful way of generating and validating disease susceptibility estimates. The concordance between data sources we observed for specific birth cohorts confirmed that data quality was high in those years, such information is helpful for evaluating data-driven targeted vaccination. In contrast, we found that coverage and incidence data were discordant for individuals in birth cohorts between 1989–1990 and 1991–1992, which suggested that one of the two sources was inaccurate. Discordant findings can trigger further investigation and lead to improved data quality.

The use of triangulation also enabled us to determine that, despite good coverage overall, there was a high proportion of susceptible individuals among those born between 1998 and 2004 (who were aged between 15 and 22 years in 2019), even after adjusting for coverage under-ascertainment. This finding was consistent with disease incidence data, which showed that most cases and outbreaks in recent years occurred in this age group.^24^ In England, where vaccine coverage is assessed using various methods, our synthesis of data from two different vaccine coverage sources led to insights into the vaccination status of the population. This data synthesis enabled us to estimate the magnitude of opportunistic vaccination, as well as evaluate the data quality of primary care vaccination records. For example, we found that data quality was not sufficient to accurately estimate vaccine coverage among adults born abroad.

In addition to the limitations inherent in both COVER and ImmForm data, the study had several other limitations. First, coverage data for catch-up campaigns were less accurate than for routine immunization. In particular, data were not collected for the 2008 catch-up campaign (unpublished regional evaluations suggest coverage was low). Second, a London-only, catch-up campaign took place in 2004 and achieved a minimum of 24% coverage for individuals born between 1985 and 2004.^25^ This campaign was not included in our study because it was regional. Consequently, susceptibility in eligible birth cohorts may have been overestimated at the national level. Third, coverage in national catch-up campaigns was assumed to be the same in all areas and eligible birth cohorts. Fourth, although 95% of general practices contributed to ImmForm data, the proportion varied between cohorts. Moreover, only 50% of practices reported data on cohorts born before September 1995. However, as these practices were spread across the country, coverage estimates for these cohorts were unlikely to have been biased. Fifth, although COVER data included single-antigen measles vaccine for birth cohorts between 1985 and 1987, ImmForm data did not. This discrepancy may explain why estimated coverage in these birth cohorts was lower for ImmForm than COVER data (Fig. 2). Sixth, during the early to mid-2000s, a small number of parents opted to have their children vaccinated using a private, unlicensed, single measles vaccine.^26^ This vaccine was not included in either COVER or ImmForm data. A 2007 study involving children born in 2001 and 2002 estimated that use of this vaccine could have increased coverage for all measles-containing vaccines by around 2% in individuals born in the early 2000s.^26^

When high-quality census data that can be linked to age-specific disease susceptibility estimates are available, the number of susceptible individuals currently in the population can be deduce, thereby enabling the size and timing of future outbreaks to be modelled. Data triangulation can improve the accuracy and precision of coverage estimates, which is vital in areas where coverage is high, and increase confidence in data. In contrast, a recent study that used incomplete and inaccurate information overestimated the number of susceptible individuals in England by a factor of 1.8.^27^

Our analytical approach involved only routinely available data sources, which are not exclusive to the United Kingdom or other high-income countries.^28^ Any country that routinely records disease incidence and vaccine coverage could consider a similar approach for measles and other diseases. However, the value of the information produced will depend on the accuracy and precision of the data available and on knowledge of how data quality varies over time. A comprehensive report on improving immunization data quality and use that was recently presented to WHO’s Strategic Advisory Group of Experts on immunization is available online.^1^ The report provides strategic guidance on improving data at the national level. In countries where data on individual years are not available, wider age ranges could be considered. Although the resulting insights would be less detailed, the analysis would still be useful for validating coverage and surveillance data and for identifying susceptible age groups. Currently, WHO is planning to publish a framework for, and guidance on, data triangulation to help countries routinely adopt the approach.^1^ One alternative to using routine data sources is to conduct much costlier and resource-intensive seroprevalence studies. In England, where the quality of both coverage and incidence data is relatively high, the added value of seroprevalence studies is limited. Current arrangements rely on residual blood samples from hospitals, which may not be representative of the general population, particularly for younger age groups.

Although national estimates of disease susceptibility can help identify at-risk birth cohorts, they may not reflect inequalities at the local level. In England, vaccine coverage varies by ethnicity, social deprivation and geographical location.^29^ Consequently, coverage is heterogeneous and the burden of measles and rubella falls disproportionately on specific communities.^30^^,^^31^ Herd immunity extends the benefits of national immunization programmes to unvaccinated individuals, thus intrinsically reducing inequalities, but its impact will depend on local and overall vaccine coverage and population mixing patterns. When a large number of unvaccinated individuals live in close proximity, their community becomes vulnerable to outbreaks. Better vaccine coverage across the whole population should be accompanied by targeted efforts to assess the risk, specific needs and characteristics of undervaccinated communities and, thereby, close any gaps in immunity.^32^

In conclusion, triangulating existing data sources on routine vaccination coverage and vaccine-preventable disease surveillance can generate new insights into a population’s level of immunity and help prioritize public health actions aimed at closing gaps in immunity. Use of this approach in England helped establish that, despite achieving high MMR vaccine coverage, measles susceptibility in particular age cohorts was sufficiently high to sustain disease transmission. Nevertheless, susceptibility in all population subgroups cannot be estimated using routine data sources alone. Ad hoc studies are needed for undervaccinated groups, such as adults born abroad. Triangulating coverage and incidence data, in particular, is a useful way of maximizing the quality of data on vaccine-preventable diseases and should be used more widely. Forthcoming guidance from WHO and its partners on the triangulation of data from the Expanded Programme on Immunization will help countries improve data use and quality and, ultimately, help control vaccine-preventable diseases.
